# At-risk registers integrated into primary care to stop asthma crises in the UK: cluster randomised controlled trial with economic evaluation

**DOI:** 10.1136/thorax-2025-223973

**Published:** 2026-04-15

**Authors:** Andrew M Wilson, Stanley D Musgrave, Jane R Smith, Michael Noble, Allan B Clark, Susan Stirling, Polly-Anna Ashford, Garry Barton, Chris Griffiths, Hilary Pinnock, David B Price, Aziz Sheikh, Samantha Walker

**Affiliations:** 1 Norwich Medical School, University of East Anglia, Norwich, UK; 2 Norfolk and Norwich University Hospital, Norwich, UK; 3 Institute of Health Research, University of Exeter Medical School, Exeter, UK; 4 Norwich Clinical Trials Unit, University of East Anglia, Norwich, UK; 5 Centre for Primary Care and Public Health, Queen Mary, University of London, London, UK; 6 Nuffield Department of Primary Care Health Sciences, University of Oxford Division of Public Health and Primary Health Care, Oxford, UK; 7 Asthma UK Centre for Applied Research, Usher Institute of Population Health Sciences and Informatics, The University of Edinburgh, Edinburgh, UK; 8 Allergy and Respiratory Group, University of Edinburgh, The University of Edinburgh, Edinburgh, UK; 9 Academic Primary Care, University of Aberdeen, Aberdeen, UK; 10 Observational and Pragmatic Research Institute, Singapore; 11 Research and Innovation, Asthma and Lung, London, UK

**Keywords:** Asthma in primary care, Health Economist

## Abstract

**Background:**

A regional trial indicated that implementing at-risk asthma registers in primary care could reduce hospital admissions. This national study assessed whether the intervention lowered asthma crisis events.

**Methods:**

This cluster randomised trial involved 275 UK primary care practices. The intervention included identifying at-risk patients, staff training, a clinical decision support system alerting practice staff to patients’ at-risk status to facilitate prompt and opportunistic care, and ongoing support. Control practices continued with standard care. Patients (n=10 945) were included if identified as at-risk, unless they declined data sharing. Routine data linked across care settings captured asthma-related crisis events (hospitalisations, accident and emergency visits or death), asthma care indicators and healthcare costs over 12 months.

**Results:**

Complete data were available from 185 practices (6207 patients), with exclusions mainly due to record linkage issues. Crisis events occurred in 7.2% of control versus 6.3% of intervention patients (OR 0.82, 95% CI 0.66 to 1.03, p=0.09). Individual components of the composite outcome showed similar, non-significant reductions. The use of systemic corticosteroids for asthma attacks had an OR of 1.18 (95% CI 0.99 to 1.41, p=0.07); personalised asthma action plans, OR 1.05 (95% CI 0.78 to 1.42, p=0.74); inhaler technique assessments, OR 1.13 (95% CI 0.93 to 1.38, p=0.23). Economic analysis estimated the intervention was cost-effective, with average annual National Health Service costs £306 lower in the intervention group.

**Conclusion:**

This trial did not provide sufficient evidence to show that the establishment and integration of at-risk registers for asthma in primary care reduces asthma-related crisis events for people with at-risk asthma, but there was some indication of benefit.

**Trial registration number:**

ISRCTN95472706.

**WHAT IS ALREADY KNOWN ON THIS TOPIC**

- Risk stratification is recommended for people with asthma, but methods of implementing this in primary care are unknown. The ARRISA (At-Risk Registers in Severe Asthma) approach, undertaken in a regional study, involves identifying those at-risk patients, flagging the records so an alert appears when they are opened, training staff on actions to take including facilitating prompt and opportunistic care, and supporting the practices.

**WHAT THIS STUDY ADDS**

- This study showed that an online trial of practice education with outcomes captured from routine data is possible. However, we did not show that our approach resulted in a statistically significant reduction in asthma crisis events (asthma-related hospitalisations, accident and emergency visits or death).

**HOW THIS STUDY MIGHT AFFECT RESEARCH, PRACTICE OR POLICY**

- We cannot recommend that at-risk registers should be established and integrated within primary care to reduce asthma-related, but we showed some indication of benefit: there was a suggestion of improved aspects of care, and the intervention was estimated to be cost-effective.

## Background

 5% of the world’s population has active asthma,^1^ and 90% of asthma deaths and 70% of hospitalisations are preventable.^2^ However, the risk of poor outcomes varies across asthma populations^3^ and methods of identifying those at high risk of an asthma attack (referred to as ‘at-risk asthma patients’), and improving care pathways to support them in primary care is therefore required.^4^

Our intervention is a primary care-based practice-level initiative comprising the generation of an at-risk asthma register at general practitioner (GP) practices,^5^ the programming of an alert or flag to appear whenever the electronic health record (EHR) of an at-risk individual is opened, staff training regarding actions to take on seeing the flag and practice support. In a regional cluster randomised controlled trial (cRCT), there was no reduction in the number of moderate–severe asthma attacks over 12 months; however, this composite outcome masked a halving of asthma hospitalisations and a 30% increase in prednisolone prescriptions for asthma attacks at intervention practices.^6^ The objective of this study was to determine whether our approach reduced asthma crisis events (asthma-related accident and emergency (A&E) attendances, hospitalisations and deaths) over a 12-month period when undertaken as a national cRCT.

## Methods

### Design

This was a two-arm pragmatic cRCT trial with health economic evaluation comparing usual care (control) to a complex, primary care practice-level intervention. The trial outline is shown in figure 1, and the protocol is published.^7^ Practice randomisation occurred between 1 September 2015 and 1 April 2018.

**Figure 1:**
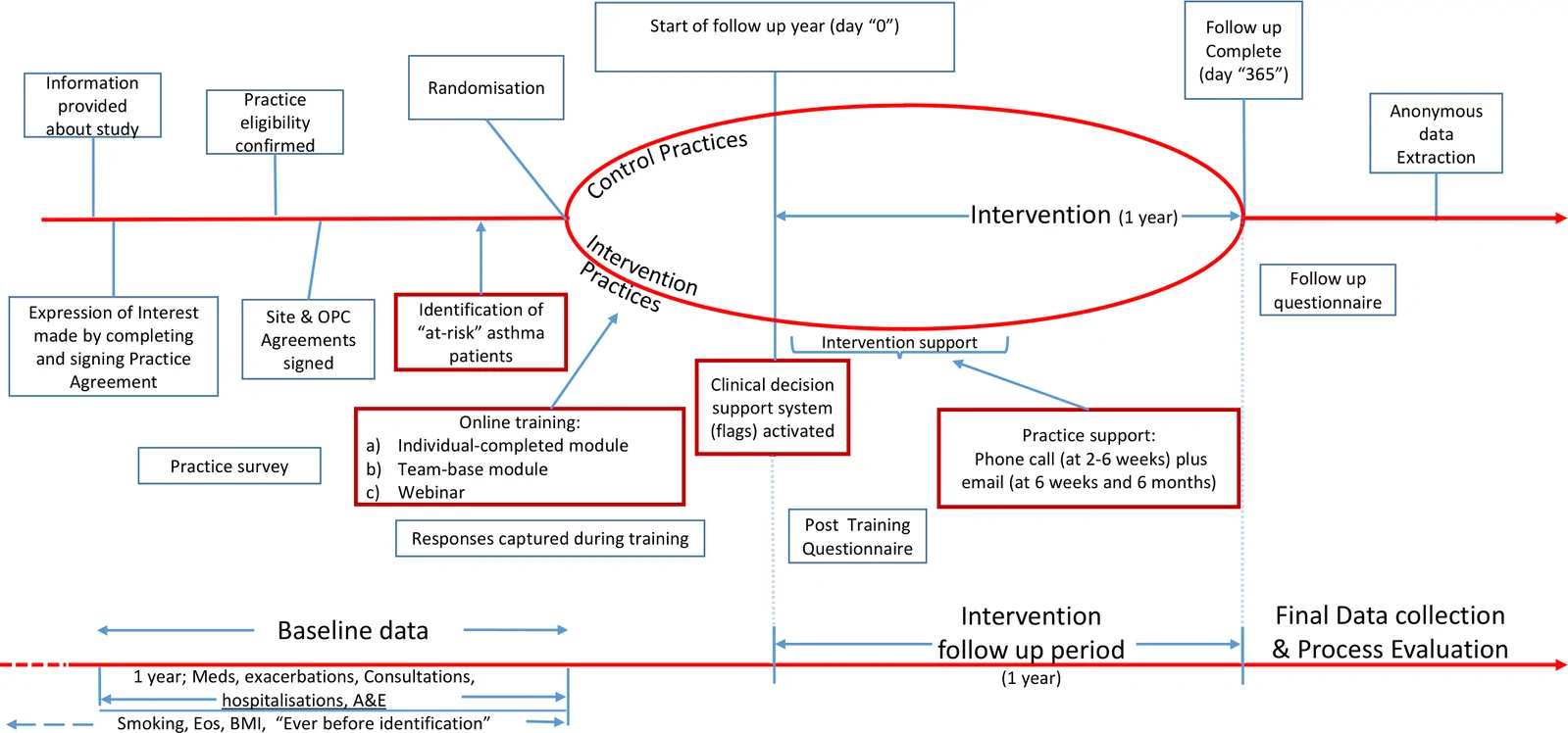
Study design. Red box represents the intervention. A&E, accident and emergency; BMI, body mass index; Eos: blood eosinophil count. OPC, Optimum Patient Care.

Routinely available data from patient EHRs were captured at the end of the trial. Optimum Patient Care (https://optimumpatientcare.org/) undertook primary care data extraction and, following Confidentiality Advisory Group (CAG) approval (8 April 2019, 18/CAG/0185), data were forwarded via Harvey Walsh (www.harveywalsh.co.uk) to National Health Service (NHS) England, who provided primary and secondary care data to Norwich Clinical Trials Unit (NCTU). For Scottish and Welsh practices, record linkage between primary and secondary care data was undertaken pseudoanonymously by Public Health Scotland and Digital Health and Care Wales, respectively. Transfer of files to NHS Digital occurred on 13 December 2021 but was incomplete, and a second transfer occurred on 5 April 2023. Secondary care data were available to the trial team on 1 August 2023.

### Setting

The setting was primary care practices within the UK, with health economic analysis from the perspective of the NHS.

### Participants

The clusters were primary care practices in the UK willing to sign agreements to participate in the trial, who did not already flag or otherwise prospectively target care towards patients with at-risk asthma on a formal practice-wide basis and were not undertaking research that might influence the trial outcome. Individual participants were patients with at-risk asthma identified using a validated algorithm^5^ and search coding (described below). In addition, data from all patients coded as having asthma (referred to as ‘all-asthma patients’) were obtained for secondary analysis. Patients with recorded refusal for use of anonymous data in research were excluded.

### Intervention

Practices were randomised to intervention or control groups, in a 1:1 ratio, based on a computer-generated randomisation code prepared by the trial statistician, with stratification for practice software (Vision/EMIS/SystmOne) and presence of an asthma diploma-trained nurse at the practice (yes/no) at the time of randomisation. Practices were informed of their allocation by email from the NCTU. The intervention was not blinded at a cluster level. Practice involvement and patients’ at-risk status were discussed at an individual level as deemed appropriate by their practice team.

#### Intervention practices

Our complex, practice-level intervention had four main components:

##### Establishment of register: identification of patients with at-risk asthma

Registers were created by applying our previously published, peer-reviewed and externally validated algorithm for identifying the 7% of individuals at highest risk of asthma-related crisis events (to achieve adequate numbers per cluster) to routine EHR data at each practice.^5^ It included factors such as previous hospitalisation, age, body mass index, smoking and blood eosinophilia and had adequate performance characteristics with 18 people needing to be placed on the register to identify one admission.^5^ Practices received a manual with instructions for identifying at-risk patients by applying search codes specific to their software system and setting up the register by placing a trial-specific Read code in each patient’s EHR.

##### Clinical decision support system

The clinical decision support system consisted of an alert or flag which appeared on the EHR whenever an identified person with at-risk asthma made contact with any member of the practice team, or their clinical record was opened. The wording of the flag, which could not be disabled, was chosen by the practice during the training (see below). The flagging went live after completion of the training.

##### Practice-wide, internet-based training

We developed a practice-wide web-based training package based on the face-to-face intervention in our previous study.^6^ Each practice nominated a practice ‘champion’ and representative from each staff discipline, for example, nurse, GP, receptionist, dispenser/pharmacist, to undertake role-specific training and disseminate key messages among their team.

The training comprised an individually completed module to explain, with self-reflection, the current problem and the intervention using a fictitious case vignette. This was followed by a team-based module, to agree and complete an action plan documenting specific actions for each type of practice staff to take when a flag was seen on the EHR of an at-risk patient, plus a plan for disseminating this to other staff. For example, actions might include prioritising appointments and clinical contact to permit early treatment of asthma attacks, ensuring guideline-based optimal care including smoking cessation advice, inhaler technique assessment and providing opportunistic support, education and treatment modification for asthma during contacts for non-asthma-related issues if appropriate. Practice representatives also attended a webinar with the trial GP lead (MN) and other practices to share experiences and ideas and refine their action plans accordingly.

##### Practice support

The practice champion received a phone call 4 weeks after the date when the flag was activated. Practice staff representatives received emails containing links to reminder videos at 6 weeks and 6 months. Staff also had access to a list of frequently asked questions and a helpline manned by an unblinded information technology (IT) technician for support with technical issues or other queries for the duration of the trial.

### Control practices

Patients with at-risk asthma were identified in the control practices using the same algorithm,^5^ but no visible flags were added to records, and staff remained blind to identified patient details. They continued to provide standard usual care based on the contemporaneous asthma guidelines^8^ and Quality and Outcome Framework asthma recommendations.

### Outcomes

#### Clinical outcomes

The primary outcome was the percentage of at-risk patients with an asthma-related crisis event. Secondary outcomes included (1) percentage of asthma-related crisis events for all patients with asthma, (2) time to first and rate of asthma-related crisis event(s), (3) individual components of the composite crisis event outcome, for both at-risk and all-asthma populations and (4) asthma control by scoring the ‘Royal College of Physicians 3 questions for asthma’^9^ for at-risk patients. Process of care outcomes included asthma medication, written asthma action plans, peak flow diaries, inhaler technique assessments, smoking cessation or influenza vaccinations. EHR-derived outcomes included ‘did not attends’ and medication adherence.^10^ Health economics assessed total NHS costs per crisis event. All data were patient-level.

### Sample size

A sample size of 8204 patients from 235 practices was estimated to provide 90% power to detect a difference in primary outcome from 7% to 5% (effect size of 0.3), assuming a cluster size of 35 and an intraclass correlation coefficient (ICC) of 0.01. We estimated that less than 10% of patients would leave the practice^11^ and there would be 100% data linkage.

### Data analysis

The comparison of intervention and control arms followed the intention-to-treat principle for both (1) patients identified as ‘at risk’ and (2) all patients with asthma, with analyses conducted separately for each group. We summarised baseline characteristics and outcomes with descriptive statistics, using the number and percentage for binary and categorical outcomes, the mean and SD for outcomes assumed to be normally distributed, and the median and IQR for ordinal outcomes. The primary outcome was analysed on an individual level using a logistic regression model with random effects (to allow for the account for clustering of patients by practice) and fixed effects (as per the stratification factors), this is the set of covariates for the ‘minimally adjusted’ analysis. An additional analysis was undertaken adjusting for the presence or absence of an A&E attendance or hospitalisation in the 12 months prior to the study period, using the same model but including a term for each of these. Estimated ORs with 95% CIs were reported, along with the ICC and its CI.

Similar models were used for the number of events using Poisson regression, with random and fixed effects, additionally including the number of days of follow-up and estimating the incidence rate ratio, and time to first event using Cox proportional hazards regression, with the same covariates and a random practice effect estimating a HR. Analyses were not performed when event counts were too low for reliable estimation. Secondary outcomes were analysed in a similar fashion with logistic regression for binary outcomes and Poisson regression for counts. Smoking cessation outcomes included people who smoked at baseline only. Analyses of short-acting beta agonist (SABA) and inhaled corticosteroid prescription proportions excluded individuals without relevant prescriptions. For the primary outcome, we assessed the sensitivity of the model assumptions using a bootstrap for the primary outcome. The bootstrap assessed the sensitivity of the results to the assumed distribution. We did not analyse residuals as there were only categorical variables in the minimally adjusted primary analysis. For Poisson regression models, overdispersion was also checked by comparing the Bayesian information criteria (BIC) values to a negative binomial regression model; the model with the smaller BIC value was used. The factors adjusted for in the models were specified prior to the analysis being undertaken based on design factors and clinical opinion.

Using the same models as above (logistic regression for binary outcomes and Poisson regression for counts), the inclusion of an interaction term was used to conduct additional post hoc subgroup analyses, not prespecified in the statistical analysis plan and as standard in RCTs, was not corrected for multiple testing, determined the effect of the outcomes based on: (1) rural versus urban practice, (2) practice software (EMIS/SystmOne/Vision), (3) practice size (split by median number of registered patients), (4) the proportion of patients identified as at-risk (by median), (5) a composite score of practice baseline asthma management strategies (≤5 vs >5 of 10 total strategies), (6) practice knowledge of National Review of Asthma Deaths (low vs moderate/high), (7) pre-existing use of a search strategy for identifying at-risk patients by practice and (8) eosinophil count (≤0.3 vs >0.3).

Data were missing in 76 practices due to the lack of linkage to secondary care data or the unavailability of GP practice data; the decision was made not to impute data as the data were missing for the whole practice. Multicollinearity was not assessed.

All tests used a 5% two-sided significance level. Analyses were performed using Stata V.18.0 or higher.

### Economic evaluation

An economic evaluation was undertaken to estimate the cost-effectiveness of the intervention, compared with usual care, as a within-trial analysis (1-year trial follow-up period, no discounting). As informed by associated guidance,^12 13^ costs were estimated from the viewpoint of the UK NHS at 2021/2022 financial year levels.

#### Intervention-related costs

Time associated with At-Risk Registers in Severe Asthma (ARRISA)-UK intervention components was estimated using a combination of automatically logged data (from staff training modules, online webinars and ongoing support such as helplines) and questionnaires completed by practice champions. To assess any additional impact on primary care, all staff who registered for the individual learning module were asked at 1 year follow-up how their total time spent with ARRISA-UK patients had changed compared with the previous year. Unit costs (online supplemental file 1) were applied to each component time, and the resulting costs were summed to estimate the total intervention-related cost. This total was then divided by the number of at-risk ARRISA-UK patients in the intervention practices.

#### Other NHS costs

The mean per patient hospital admission and A&E attendance and asthma medication-related costs were calculated.

#### Total NHS costs

Other NHS costs were the only costs estimated for control patients. For intervention patients, total NHS costs were estimated by summing the total ARRISA-UK intervention-related costs and other NHS costs.

#### Outcomes

The main outcome for the economic analysis was the per patient mean number of asthma-related crisis events over the 12-month follow-up period.

#### Analyses

The mean total NHS costs and outcomes were estimated for at-risk patients in both groups, and the mean difference between the groups estimated the mean incremental cost and benefit associated with the intervention. If total per patient mean costs were estimated to be lower in the intervention group, and the benefit was also estimated to be higher, then the intervention would be estimated to be cost-effective.^14^

## Results

### Recruitment

A total of 539 practices (figure 2) expressed an interest in participating in the trial and 275 practices (table 1), managing 10 945 patients with at-risk asthma (table 2), were randomised. Data from 185 (67%) practices (6207 patients) were available for a complete case analysis, mostly due to difficulties with data extraction and record linkage between primary and secondary care data.^15^ The groups were similar in terms of both practice and patient-based characteristics (table 1).

**Figure 2:**
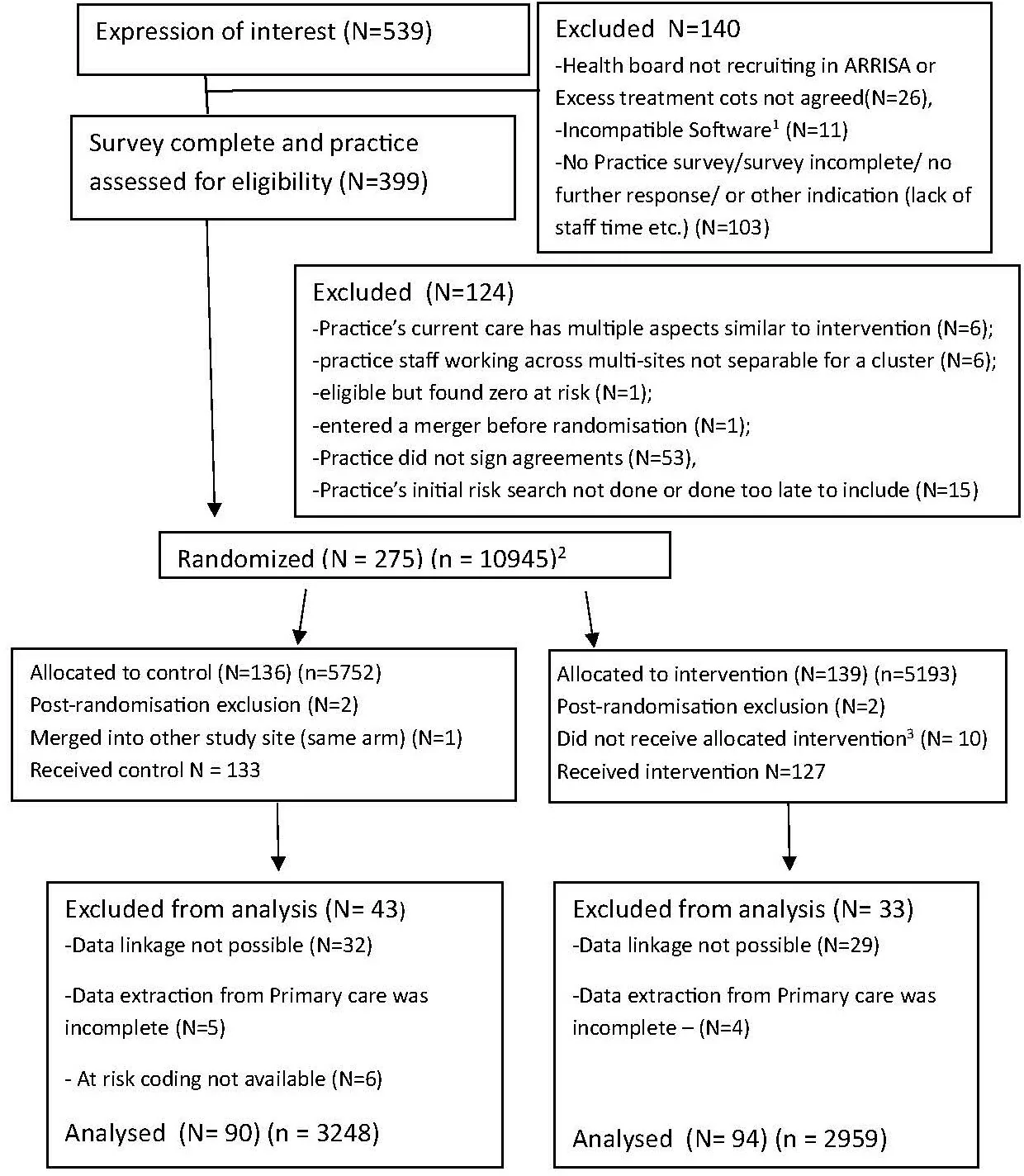
Flow of participants (N=practices, n=patients) for at-risk patients. ^1^EMISPCS (N=9) Microtest (N=2) ^2^Information on the number of asthma and atrisk patients within practices at early stages of the study is only available as an estimate specific counts of those with data available for analysis were only confirmed after final data extraction and matching at the end of the study ^3^Minimum training required to proceed with intervention was at least one member completing Module 1, Module 2 and webinar and coding of at-risk patients. ARRISA, At-Risk Registers in Severe Asthma.

**Table 1:** Baseline practice characteristics

	Randomised		Available for the primary outcome	
	Control N=136	Intervention N=139	Control N=90	Intervention N=94
Mean (SD) practice population	9887.14 (5418.23)	9351.53 (4799.08)	9844.98 (5358.68)	9087.76 (4775.37)
Mean (SD) number of patients per practice identified as at-risk at study initiation	42.29 (29.94)	37.36 (28.31)	41.81 (30.08)	37.07 (30.37)
Mean (SD) percentage of patients with asthma per practice identified as at-risk at study initiation	6.58 (2.86)	6.26 (2.67)	6.61 (2.83)	6.26 (2.91)
Clinical computer software used at time of randomisation (number of practices (%)):				
EMIS WEB	66 (48.5)	66 (47.5)	44 (49)	45 (48)
SystmOne	52 (38.2)	54 (38.9)	40 (44)	39 (41)
VISION+others	18 (13.2)	19 (13.7)	6 (7)	10 (11)
No of practices with asthma/resp specialist GP	38 (27.9)	35 (25.2)	23 (26)	20 (21)
Urban	104 (76.5)	103 (74.1)	69 (77)	69 (73)
Rural	32 (23.5)	36 (25.9)	21 (23)	25 (27)
Number (%) of practices with one or more asthma diploma-trained nurses	95 (69.9)	96 (69.1)	62 (69)	63 (67)
Number (%) of dispensing practices	38 (27.9)	35 (25.2)	23 (26)	25 (27)
No (%) of practices with asthma service led by:				
GP	33 (24.3)	29 (20.9)	19 (21)	19 (20)
Nurse	100 (73.5)	100 (71.9)	69 (77)	68 (72)
Other	2 (1.5)	3 (2.2)	2 (2)	3 (3)
None	1 (0.7)	7 (5.0)	0 (0)	4 (4)
No (%) of practices using the following search strategies for identification and management of patients with at-risk asthma				
Regular searches involving ID of a subgroup of people with at-risk asthma	25 (18.4)	29 (20.9)	13 (14)	19 (20)
Formal at-risk asthma register	18 (13.2)	29 (20.9)	8 (9)	17 (18)
Flagging of electronic health records (visible at every patient contact)	45 (33.1)	44 (31.7)	26 (29)	27 (29)
Prioritisation of appointments for patients with at-risk asthma	38 (27.9)	31 (22.3)	29 (32)	17 (18)
Follow-up of non-attenders	69 (50.7)	73 (52.5)	41 (46)	48 (51)
Mechanisms for identifying poor adherence to medications	74 (54.4)	78 (56.1)	47 (52)	50 (53)
Increased frequency of review for at-risk patients (ie, more than one QoF annual review)	79 (58.1)	70 (50.4)	49 (54)	45 (48)
Increased proactive provision and review of self-management plans for at-risk patients	54 (39.7)	57 (41.0)	35 (39)	36 (38)
No (%) of practices with no specific strategies for identification and management of patients with at-risk asthma	31 (22.8)	30 (21.6)	24 (27)	23 (24)
Other strategies	7 (5.1)	7 (5.0)	5 (6)	6 (6)
No (%) of practices using a proforma for routine asthma clinic consultations	127 (93.4)	135 (97.1)	84 (93)	91 (97)
Degree of confidence in strategies for managing at-risk/difficult asthma: no (%) of practices				
Not confident at all	1 (0.7)	2 (1.4)	1 (1)	2 (2)
Not very confident	8 (5.9)	11 (7.9)	5 (6)	10 (11)
Neutral	51 (37.5)	54 (38.8)	37 (41)	35 (37)
Confident	69 (50.7)	65 (46.8)	43 (48)	44 (47)
Very confident	7 (5.1)	7 (5.0)	4 (4)	3 (3)

GP, general practitioner; N, number; QoF, Quality and Outcomes Framework; resp, respiratory.

**Table 2:** Baseline patient characteristics

	Patients with at-risk asthma	
	Control (n=4300)	Intervention (n=4180)
Age (years), mean (SD)	59.8 (21.2)	59.43 (21.6)
Gender		
Female, n (%)	2867 (66.7%)	2789 (66.7%)
Smoking status		
Current, n (%)	1046 (25.1%)	999 (24.8%)
Ex, n (%)	1491 (35.8%)	1469 (36.5%)
Never, n (%)	1633 (39.2%)	1561 (38.7%)
Body mass index category		
Underweight (<18.5 kg/m^2^)	326 (7.6%)	334 (8.0%)
Healthy (18.5–25 kg/m^2^)	988 (23.0%)	938 (22.4%)
Overweight (25–30 kg/m^2^	899 (20.9%)	878 (21.0%)
Obesity class 1 (30–35 kg/m^2^)	831 (19.3%)	770 (18.4%)
Obesity class 2 (35–40 kg/m^2^)	485 (11.3%)	461 (11.0%)
Obesity class 3 (≥40 kg/m^2^)	394 (9.2%)	357 (8.5%)
Missing	377 (8.8%)	442 (10.6%)
Number of asthma-related crisis events, median (IQR)	0 (0–0)	0 (0–0)
0	2893 (88.07%)	2773 (87.26%)
1	247 (7.52%)	238 (7.49%)
2	91 (2.77%)	98 (3.08%)
More than 2	54 (1.64%)	69 (2.17%)
Number of asthma-related A&E attendances, median (IQR)	0 (0–0)	0 (0–0)
0	3108 (94.61%)	2982 (93.83%)
1	139 (4.23%)	148 (4.66%)
2	23 (0.70%)	33 (1.04%)
More than 2	15 (0.46%)	15 (0.47%)
Number of asthma-related hospitalisations, median (IQR)	0 (0–0)	0 (0–0)
0	2966 (90.29%)	2861 (90.03%)
1	257 (7.82%)	236 (7.43%)
2	40 (1.22%)	49 (1.54%)
More than 2	22 (0.67%)	32 (1.01%)
Good asthma control, n (%)		
No	329 (16.1%)	303 (16.%)
Yes	1716 (83.9%)	1511 (83.3%)
Number of short-acting bronchodilator prescriptions, median (IQR)	5.0 (2.0–10.0)	5.0 (2.0–10.0)
0	305 (7.1%)	272 (6.5%)
1	439 (10.2%)	446 (10.7%)
2	407 (9.5%)	390 (9.3%)
More than 2	3149 (73.2%)	3072 (73.5%)
Number of long-acting bronchodilator prescriptions, median (IQR)	0 (0–0)	0 (0–0)
0	4132 (96.1%)	4019 (96.2%)
1	30 (0.7%)	26 (0.6%)
2	12 (0.3%)	17 (0.4%)
More than 2	126 (2.9%)	118 (1.8%)
Number of LABA (mono only) prescriptions, median (IQR)	0 (0–0)	0 (0–0)
0	4178 (97.2%)	4062 (97.2%)
1	40 (0.9%)	51 (1.2%)
2	30 (0.7%)	17 (0.4%)
More than 2	52 (1.21%)	50 (1.2%)
LABA		
0	1251 (29.1%)	1254 (30.0%)
1	167 (3.9%)	127 (3.0%)
2	197 (4.6%)	161 (3.9%)
More than 2	2685 (62.4%)	2.638 (63.1%)
Prescriptions of systemic corticosteroids for asthma attacks and antibiotic lower respiratory tract infections, median (IQR)	0 (0–1)	0 (0–1)
0	2346 (54.6%)	2168 (51.9%)
1	1196 (27.8%)	1176 (28.1%)
2	484 (11.3%)	503 (12.0%)
More than 2	274 (6.4%)	333 (8.0%)
Prescriptions of inhaled corticosteroids, median (IQR)	7 (3–11)	6 (3–11)
0	389 (9.1%)	488 (11.7%)
1	237 (5.5%)	200 (4.8%)
2	287 (6.7%)	233 (5.6%)
More than 2	3387 (78.8%)	3259 (78.0%)
Record of written personalised asthma action plans n (%)		
No	3078 (71.6%)	2820 (67.5%)
Yes	1222 (28.4%)	1360 (32.5%)
Record of patient self-monitoring with peak flow diaries n (%)		
No	4266 (99.2%)	4173 (99.8%)
Yes	34 (0.8%)	7 (0.2%)
Inhaler technique assessments recorded, n (%)		
No	2159 (50.2%)	2143 (51.3%)
Yes	2141 (49.8%)	2037 (48.7%)
Smoking cessation advice or medications given, people who smoke only, n (%)		
No	151 (14.4%)	160 (16.0%)
Yes	895 (85.6%)	839 (84.0%)
Influenza vaccinations, n (%)		
No	1569 (36.5%)	1517 (36.3%)
Yes	2731 (63.5%)	2663 (63.7%)
Number of ‘did not attend’ appointments in primary care, median (IQR)		
0	2022 (47.0%)	2066 (49.4%)
1	1036 (24.1%)	969 (23.2%)
2	490 (11.4%)	480 (11.5%)
More than 2	752 (17.5%)	665 (9%)
Eosinophil count		
No count	1500 (34.9%)	1491 (35.7%)
≤0.3	1997 (46.4%)	1941 (46.4%)
>0.3	803 (18.7%)	748 (17.9%)

Baseline data is available on less than those randomised due to incomplete data extraction from primary care.

A&E, accident and emergency; LABA, long active beta agonist; n, number.

### Primary outcome

For the at-risk population, 7.2% (235/3248) of people in the control practices versus 6.3% (185/2959) in the intervention practices experienced an asthma-related crisis event with an OR of 0.87 (95% CI 0.69 to 1.09, p=0.22) when adjusted for the stratification variables. After adjusting for the presence or absence of baseline hospitalisation and A&E attendance, the OR was 0.82 (95% CI 0.66 to 1.03, p=0.09). The bootstrap analysis gave very similar results: 0.87 (95% CI 0.70 to 1.08, p=0.21) for the minimally adjusted and 0.82 (95% CI 0.66 to 1.02, p=0.07) for the adjusted.

There was no evidence of multicollinearity.

### Secondary outcomes

There were no significant differences in the rate of, or time to, first crisis events as a composite outcome or the separate components, nor was there for asthma control between the intervention and control arm for the at-risk asthma population (table 3). There were only three all-cause deaths (one in the control and two in the intervention groups) for the at-risk asthma population, and therefore death was not analysed separately.

**Table 3:** Results comparing intervention versus control practices for primary and secondary outcomes: at-risk population

	Control	Intervention	Minimally adjusted	P value	Adjusted for baseline	P value
	(n=3248; N=90)	(n=2959; N=94)	OR (95% CI)		OR (95% CI)	
Primary outcome: one or more asthma-related crisis event*	235 (7.2%)	185 (6.3%)	0.87 (0.69 to 1.09)	0.220	0.82 (0.66 to 1.03)	0.088
Time to first asthma-related crisis event (years)†	Incidence rate 0.08	Incidence rate 0.06	HR 0.84 (0.67 to 1.06)	0.145	N/A	
Time to first asthma-related A&E attendance†	Incidence rate 0.04	Incidence rate 0.03	HR 0.84 (0.61 to 1.16)	0.282	N/A	
Time to first asthma-related hospitalisation†	Incidence rate 0.06	Incidence rate 0.05	HR 0.86 (0.68 to 1.08)	0.184	N/A	
Asthma-related A&E*	129 (4.0%)	96 (3.2%)	0.85 (0.61 to 1.19)	0.351	0.82 (0.59 to 1.13)	0.214
Asthma-related hospitalisation*	181 (5.6%)	140 (4.7%)	0.85 (0.67 to 1.08)	0.184	0.81 (0.64 to 1.03)	0.082
Rate of asthma crisis event (per year)‡§¶	0.19 (1.07)	0.15 (1.07)	0.87 (0.61 to 1.23)	0.425	0.79 (0.58 to 1.07)	0.124
Rate of asthma-related A&E (per year)‡§¶	0.06 (0.40)	0.06 (0.50)	0.97 (0.63 to 1.49)	0.891	0.91 (0.62 to 1.34)	0.640
Rate of asthma-related hospitalisation (per year)‡§¶	0.11 (0.78)	0.10 (0.72)	0.89 (0.63 to 1.25)	0.491	0.80 (0.60 to 1.07)	0.130
Good asthma control*	1653 (86.91%) (n=1902)	1419 (84.72%) (n=1675)	0.39 (0.07 to 2.15)	0.279		

* Logistic model.

† Cox model.

‡ Mean (SD).

§ Incident rate ratio.

¶ Negative Binomial model.

A&E, accident and emergency; N/A, not assessed.

For the indicators of asthma care outcomes for the at-risk population (table 4), the rate ratio for the number of prescriptions of reliever inhalers per patient was 0.99 (95% CI 0.95 to 1.04, p=0.65), and the OR for patients with any inhaled corticosteroid prescription was 0.77 (95% CI 0.61 to 0.97, p=0.026), any systemic corticosteroid therapy for an asthma attack was 1.18 (95% CI 0.99 to 1.41, p=0.065), any personalised asthma action plan was 1.05 (95% CI 0.78 to 1.42, p=0.74) or any inhaler technique assessment was 1.13 (95% CI 0.93 to 1.38, p=0.23). There were no differences between groups in any other process of care.

**Table 4:** Comparison of process of care outcomes between treatment groups for at-risk asthma population

	Control	Intervention	Minimally adjusted		Effect Size (95% CI)	P value
	(n=4204)	(n=4103)	Effect Size (95% CI)	P value		
Any prescriptions of systemic corticosteroids for asthma attack and antibiotic treated LRTI	1113 (26.47%)	1233 (30.05%)	1.22 (0.99 to 1.50)*	0.057	1.181*(0.99 to 1.41)	0.065
Rate of SABA prescriptions issued	6.14 (5.52)	6.15 (5.67)	1.042 (0.96 to 1.12)^∧^	0.324	0.992^∧^ (0.95 to 1.04)	0.651
Rate of prescriptions of systemic corticosteroids for asthma attack and antibiotic treated LRTI	0.45 (1.01)	0.52 (1.05)	1.172^∧^ (0.98 to 1.41)	0.084	1.142^∧^ (0.98 to 1.33)	0.083
Any inhaled corticosteroid prescriptions	3601 (85.66%)	3315 (80.79%)	0.821* (0.63 to 1.05)	0.119	0.771* (0.61 to 0.97)	0.026
Rate of inhaled corticosteroid prescriptions	7.26 (8.24)	6.75 (6.12)	0.972^∧^ (0.87 to 1.07)	0.484	0.972^∧^ (0.90 to 1.04)	0.406
Any LABA	95 (2.26%)	99 (2.41%)	1.091* (0.78 to 1.53)	0.604	1.181* (0.80 to 1.72)	0.404
LABA monotherapy	65 (1.6%)	63 (1.5%)	1.041* (0.71 to 1.50)	0.853	1.061* (0.70 to 1.60)	0.798
Any LABA	2857 (68.0%)	2676 (65.2%)	0.911* (0.74 to 1.11)	0.356	0.801* (0.64 to 1.01)	0.058
Rate of LABA prescriptions	5.7 (6.0)	5.6 (6.1)	0.972^∧^ (0.86 to 1.09)	0.616	0.962^∧^ (0.87 to 1.05)	0.378
Record of patient self-monitoring with peak flow diaries	29 (0.69%)	3 (0.07%)	3.121* (0.03 to 349.152)	0.637	3.181* (0.04 to 247.67)	0.602
Record of written personalised asthma action plans	1246 (29.64%)	1319 (32.15%)	1.081* (0.77 to 1.53)	0.653	1.051*(0.78 to 1.42)	0.736
Inhaler technique assessments recorded	1961 (46.65%)	1881 (45.84%)	1.161* (0.92 to 1.45)	0.214	1.131* (0.93 to 1.38)	0.226
Smoking cessation advice or smoking cessation medications given (restricted to people who smoke at baseline)	744 (72.94%)	723 (73.25%)	1.071* (0.80 to 1.43)	0.65	1.091* (0.83 to 1.43)	0.55
Influenza vaccination	2329 (55.40%)	2400 (58.49%)	0.961* (0.68 to 1.36)	0.833	1.031* (0.74 to 1.42)	0.881
Medication adherence	43.42 (41.65)	38.92 (43.55)	−3.87$ (−7.59 to −0.14)	0.042	−2.18$ (−4.12 to −0.24)	0.027
DNA appointments	1.29 (2.14)	1.16 (1.98)	0.992* (0.85 to 1.14)	0.844	1.012* (0.89 to 1.14)	0.884

* Odds ratio, ∧Incident rate ratio $ mean difference

DNA, ‘did not attend’; LABA, long active beta agonists; LRTI, lower respiratory tract infection; SABA, short-acting beta agonist.

ICCs are provided in the online supplemental file 1.

### Subgroup analyses

There were no differences between subgroups based on baseline practice characteristics (table 5) or baseline patient blood eosinophil count (p value for interaction 0.88, unadjusted, 0.58 adjusted).

**Table 5:** Subgroup analyses by practice characteristics for at-risk asthma population

	Control	Intervention	Minimally adjusted			Adjusted for baseline		
			OR (95% CI)	P value	P value of interaction	OR (95% CI)	P value	P value of interaction
Urban	188/2536 (7.4%)	153/2241 (6.8%)	0.94 (0.72 to 1.22)	0.642	0.535	0.87 (0.67 to 1.12)	0.278	0.507
Rural	47/712 (6.6%)	32/718 (4.5%)	0.64 (0.39 to 1.03)	0.064		0.66 (0.39 to 1.09)	0.103	
Small practice size	65/955 (6.8%)	70/1134 (6.2%)	0.92 (0.63 to 1.35)	0.682	0.778	0.87 (0.60 to 1.25)	0.446	
Large practice size	170/2293 (7.4%)	115/1825 (6.3%)	0.86 (0.65 to 1.14)	0.298		0.81 (0.61 to 1.07	0.136	0.709
Proportion of at-risk patients								
Fewer ‘at risk’	62/882 (7.0%)	64/912 (7.0%)	1.07 (0.73 to 1.56)	0.731	0.40	1.12 (0.75 to 1.67)	0.572	0.141
More ‘at risk’	173/2366 (7.3%)	121/2047 (5.9%)	0.83 (0.62 to 1.10)	0.200		0.76 (0.57 to 1.01)	0.057	
NRAD knowledge								
Nothing/very little	87 (7.1%)	67 (7.3%)	1.00 (0.71 to 1.41)	0.988	0.240	0.91 (0.64 to 1.31)	0.626	0.376
Fair amount/good amount/very familiar	148 (7.3%)	118 (5.8%)	0.79 (0.59 to 1.06)	0.115		0.78 (0.58 to 1.04)	0.094	
Strategies for managing asthma								
One or more strategy	175 (7.6%)	130 (6.4%)	0.85 (0.66 to 1.10)	0.218	0.702	0.83 (0.63 to 1.08)	0.157	0.922
No strategy	60 (6.0%)	55 (5.8%)	0.96 (0.61 to 1.50)	0.844		0.85 (0.55 to 1.30)	0.448	
Composite score of strategies for managing asthma								
Score≤5	94/1226 (7.7%)	83/1275 (6.51%)	0.88 (0.62 to 1.27)	0.503	0.99	0.83 (0.59 to 1.17)	0.293	0.799
Score>5	141/2022 (6.97%)	102/1684 (6.06%)	0.87 (0.65 to 1.16)	0.342		0.83 (0.62 to 1.11)	0.201	
EHR software								
EMIS	130/1810 (7.18%)	104/1618 (6.43%)	0.89 (0.65 to 1.22)	0.479	0.95	0.81 (0.60 to 1.09)	0.155	0.95
SystmOne	92/1249 (7.37%)	64/1053 (6.08%)	0.81 (0.57 to 1.16)	0.245		0.84 (0.57 to 1.23)	0.37	
VISION	13/189 (6.88%)	17/288 (5.90%)	1.06 (0.48 to 2.33)	0.888		1.00 (0.45 to 2.20)	0.992	
Diploma trained nurse								
No	83/1119 (7.42%)	57/796 (7.16%)	0.92 (0.61 to 1.36)	0.658	0.72	0.87 (0.59 to 1.29)	0.489	0.64
Yes	152/2129 (7.14%)	128/2163 (5.92%)	0.85 (0.65 to 1.12)	0.244		0.80 (0.61 to 1.05)	0.106	

EHR, electronic health record; NRAD, National Review of Asthma Deaths.

#### All-asthma population

The all-asthma population included 168 661 patients. They were younger, more likely to be male, to have smoked and to have better asthma control than the at-risk patients. For the primary endpoint, 1.99% in the control practices versus 1.80% in the intervention practices experienced an asthma-related crisis event (OR 0.87, 95% CI 0.69 to 1.09, p=0.24) when adjusted for the stratification variables or after adjusting for baseline hospitalisation and A&E attendance (OR 0.93, (95% CI 0.82 to 1.07), p=0.32). There was no difference between arms for any of the other secondary outcomes.

### Harms

We had no reports of adverse events, concerns or harms.

### Economic analysis

#### Costs

The total ARRISA-UK intervention-related cost was estimated to be £481 453 (equivalent to a mean per patient cost of £163 when apportioned across the 2959 ARRISA-UK patients for whom primary outcome data were available). The mean per patient other NHS costs were estimated to be £468 lower in the intervention compared with control practices. This was due to estimated mean per patient hospital admission, A&E and asthma-related medication costs being lower in the intervention arm (£2,581, £345 and £150, respectively) compared with the control group (£2,999, £379 and £167, respectively). Thus, they were estimated to more than offset the cost of the intervention, with the mean incremental total NHS cost associated with the ARRISA-UK intervention estimated to be £306 lower in the intervention arm.

#### Outcomes

The mean per patient number of asthma-related crisis events was 0.01 lower in the intervention group (0.15 per at-risk patient) compared with the control group (0.16 per at-risk patient).

#### Analyses

Total NHS costs and the number of asthma-related crisis events were both therefore estimated to be lower in the intervention compared with the control arm, suggesting that the ARRISA-UK intervention dominates/is more cost-effective than usual care as it is estimated to be both less costly and more effective.

## Discussion

### Main findings

This trial did not show a significant reduction in the percentage of people experiencing an asthma crisis compared with usual care. Likewise, there were no significant effects on A&E attendances, hospitalisation, asthma control or indicators of asthma care. However, rates of acute asthma events were numerically lower, and appropriate treatment and care processes were higher. There was no clear evidence that the intervention better aligned treatment with guidelines or improved ‘did not attend’ rates. The health economic analysis, focusing on mean values rather than statistical significance,^16^ estimated that the intervention was cost-effective.

The main strength is that this was a large trial of 275 primary care practices across the UK, using all primary care EHR software systems. The use of routine data in research is encouraged due to the inclusion of all patients, efficient data capture and potential to reduce research waste.^17^ It was a pragmatic, effectiveness trial, evaluating the delivery and operation of the intervention in real life.

The main limitation is the large number of practices for which record linkage was not obtained. The original protocol required only anonymous data for linkage, but changes in NHS Digital procedures demanded patient-identifiable data, so, after CAG approval, we had to recontract all English practices. Combined with software changes, IT updates and unusable data (eg, due to non-standard coding), this reduced our analysable sample from 10 945 to 6207 patients, lowering trial power. It also caused significant delays and there have been changes in the management of people with asthma during this time. The most important of these is the introduction of biological agents for people with severe asthma^18^ which have had a considerable effect on the rate of asthma crisis events and could potentially reduce any impact of our intervention if applied in current practice. However, the intervention includes important strategies—targeting and prioritising at-risk patients, promoting preventative care and improving communication—that remain relevant to asthma management despite new treatments.

The trial used a 12-month duration, in keeping with previous studies,^6^ as it can take 12 months to show an effect on primary care process changes.^19^ However, full implementation may take longer, or enthusiasm may decline. Given high one-off setup costs, sustained benefit over time would likely increase the estimated cost-effectiveness from a longer-term perspective.

We used a validated algorithm to identify people with at-risk asthma from routine healthcare data,^5^ despite the limitations of possible miscoding, as identifying the risk factors (severe asthma plus adverse behavioural or psychosocial factors) described in contemporaneous guidelines^8^ would have required manual review of EHR free text.^6^ Current guidelines state that risk factors include non-adherence, overuse of SABAs and A&E attendance or hospital admission. Our algorithm was developed using multiple logistic regression models applied to large primary care datasets and incorporating the known risk factors available in routine data at the time, including hospital admission, blood eosinophilia^20^ (unavailable in a third of the Asthma-UK sample), but not fraction of exhaled nitric oxide or lung function as these were not at that point routinely available in primary care. Current guidance advocates greater use of these measurements^21 22^ and recent data^23^ would suggest that these be given greater weight in the algorithm which would change those deemed to be at risk. We envisage that, in future, asthma risk may be calculated in much the same way as Cardiovascular Risk Score (QRISK) identifies those at risk of cardiovascular events^24^ and be updated in real time as patient characteristics change and new algorithms/data emerge.

A further limitation is that the health economic outcome was not based on the Quality Adjusted Life Year.^12^ Additionally, the estimated total ARRISA-UK intervention-related cost should be treated with caution, as some elements were based on staff recall rather than empirical data. Some reported values seemed particularly high, but excluding these would lower the estimated total intervention cost and not change the conclusion.

### Generalisability

This trial was based on the previous regional ARRISA trial with comparable (although online) training material, identical flagging process and similar action planning. That trial also suggested avoidance of hospital admissions due to early prednisolone therapy for asthma attacks and was estimated to be cost-effective.^6^ Indeed, an individual patient meta-analysis of the two studies provided similar non-significant results to the main trial for asthma crisis events, but with narrower confidence intervals (OR 0.82, (95% CI 0.66 to 1.01) p=0.068).

Previous studies have assessed how asthma education and organisational changes affect outcomes. The Pediatric Asthma Care Patient Outcomes Research Team study^25^ found that practice champions reduced asthma attacks but did not examine hospital admissions or crisis events. The East London Randomised Controlled Trial for High Risk Asthma trial^26^ showed that patient self-management and nurse-led reviews delayed first unscheduled care visits. However, the

Effect of an Education Programme for South Asians with Asthma and Their Clinicians: A Cluster Randomised Controlled Trial (OEDIPUS)

trial^27^ found that education for South Asian patients and their physicians did not reduce unscheduled care, suggesting that education alone may not be effective without infrastructure support. Notably, unlike ARRISA-UK, participants in these studies gave written consent, indicating voluntary participation.

We were concerned that the ARRISA-UK intervention would divert care away from the whole asthma population towards the at-risk patients.^28^ However, the number of asthma crisis events was (not significantly) lower in the intervention group when considering the whole asthma population.

Evaluation of subgroups suggests that those practices with more at-risk patients (24% reduction in events) had better outcomes than those with fewer at-risk patients (12% increase in events) and rural practices (34% reduction) had better outcomes than urban practices (13% reduction) in terms of the primary outcome. It is likely that those practices with more at-risk patients were more likely to see the flag, and the ARRISA-UK intervention is likely to become better integrated into care. Those living in urban areas may be more likely to attend A&E than contact their practice.

## Conclusion

This study did not provide sufficient evidence to show that the establishment and integration of at-risk registers for asthma in primary care reduces asthma-related crisis events for people with at-risk asthma. However, there was some indication of benefit, which became more convincing when combined with previous data. The findings from the secondary data could represent improved aspects of asthma care, and the intervention was estimated to be cost-effective. Targeting care to at-risk patients did not adversely affect the care of others. Future use in clinical practice would be facilitated by the incorporation of at-risk algorithms and flagging within EHR and potentially the use of artificial intelligence to tailor support for practice clinicians.

## Supplementary material

10.1136/thorax-2025-223973 online supplemental file 1

10.1136/thorax-2025-223973 online supplemental file 2

## Data Availability

Data are available upon reasonable request.
