# Supplementary material for: At-risk registers integrated into primary care to stop asthma crises in the UK: cluster randomised controlled trial with economic evaluation
Source: Thorax. 2026 Apr 15;81(11):e223973. doi: 10.1136/thorax-2025-223973 (PMC13691709; doi:10.1136/thorax-2025-223973)
Supplement: Supplementary file 1 [file thorax-81-11-s001.pdf]

Outcome	At-risk participants		All asthma participants	
	Minimally adjusted	Fully adjusted	Minimally adjusted	Fully adjusted
Primary	0.03 (0.01,0.08)	0.01 (0.00,0.10)	0.04 (0.03,0.06)	0.03 (0.02,0.04)
Asthma-related hospitalisation	0.01 (0.00,0.15)	0.00 (0.00,1.00)	0.02 (0.1,0.04)	0.01 (0.01,0.03)
Asthma-related adverse event	0.09 (0.04,0.17)	0.06 (0.02,0.15)	0.10 (0.07,0.14)	0.08 (0.06,0.11)
Any LABA monotherapy	0.08 (0.03,0.18)	0.00 (0.00,1.00)	0.10 (0.08,0.13)	0.04 (0.02,0.07)
Record of written personalised asthma action plans	0.36 (0.30,0.42)	0.30 (0.25,0.37)	0.49 (0.44,0.55)	0.47 (0.41,0.52)
Record of patient self-monitoring with peak flow diaries	0.78 (0.31,0.97)	0.77 (0.37,0.95)	0.62 (0.47,0.75)	0.60 (0.45,0.73)
Inhaler technique assessments recorded	0.15 (0.12,0.19)	0.11 (0.08,0.14)	0.21 (0.18,0.25)	0.17 (0.15,0.21)
Smoking cessation advice or smoking cessation medications given (restricted to smokers at baseline)	0.11 (0.07,0.18)	0.08 (0.04,0.15)	0.10 (0.08,0.12)	0.08 (0.07,0.11)
Flu vaccination	0.31 (0.26,0.36)	0.28 (0.23,0.33)	0.34 (0.30,0.39)	0.30 (0.26,0.35)
Shorting acting bronchodilator prescriptions issued	0.07 (0.06,0.09)	0.02 (0.02,0.03)	0.09 (0.07,0.10)	0.03 (0.03,0.04)
Any Prescriptions of systemic corticosteroids for asthma attacks and antibiotic treated lower respiratory tract infections	0.11 (0.08,0.14)	0.07 (0.05,0.09)	0.13 (0.10,0.15)	0.09 (0.07,0.12)
Adherence	0.08 (0.07,0.10)	0.03 (0.02,0.04)	0.06 (0.05,0.07)	0.03 (0.02,0.03)
DNA appointments	0.28 (0.23,0.34)	0.19 (0.15,0.24)	0.28 (0.23,0.33)	0.21 (0.17,0.25)
