# Supplementary material for: At-risk registers integrated into primary care to stop asthma crises in the UK: cluster randomised controlled trial with economic evaluation
Source: Thorax. 2026 Apr 15;81(11):e223973. doi: 10.1136/thorax-2025-223973 (PMC13691709; doi:10.1136/thorax-2025-223973)
Supplement: Supplementary file 2 [file thorax-81-11-s002.pdf]

At-Risk Registers Integrated into primary care to Stop Asthma crises in the UK: cluster randomised controlled trial with economic evaluation

Wilson AM, et al. Thorax 2025. DOI 10.1136/thorax-2025-223973

Creation of At-risk Asthma Register

Alert on medical records to show at-risk status

Practice staff training regarding actions on seeing alert

Practice based support

Flow of at-risk asthma participants (N = practices, n = patients)

Survey complete and practice assessed for eligibility (N=399)

Randomized (N = 275) (n = 10945)

Control (N=136) (n=5752)
Post-randomisation exclusion (N=2)
Merged into other study site (same arm) (N=1)

Intervention (N=139) (n=5193)
Post-randomisation exclusion (N=2)
Did not receive allocated intervention (N= 10)

Not analysed (N= 43)
-Data linkage not possible (N=32)
-Data extraction from Primary care was incomplete (N=5)
- At risk coding not available (N=6)
Analysed (N= 90) (n = 3248)

Not Analysed (N= 33)
-Data linkage not possible (N=29)
-Data extraction from Primary care was incomplete – (N=4)
Analysed (N= 94) (n = 2959)

	Control	Intervention	OR (95%CI)	p-value
Primary Outcome: one or more asthma-related crisis event	235 (7.2%)	185 (6.3%)	0.82 (0.66, 1.03)	0.088

1% absolute or 18% relative reduction in asthma crises

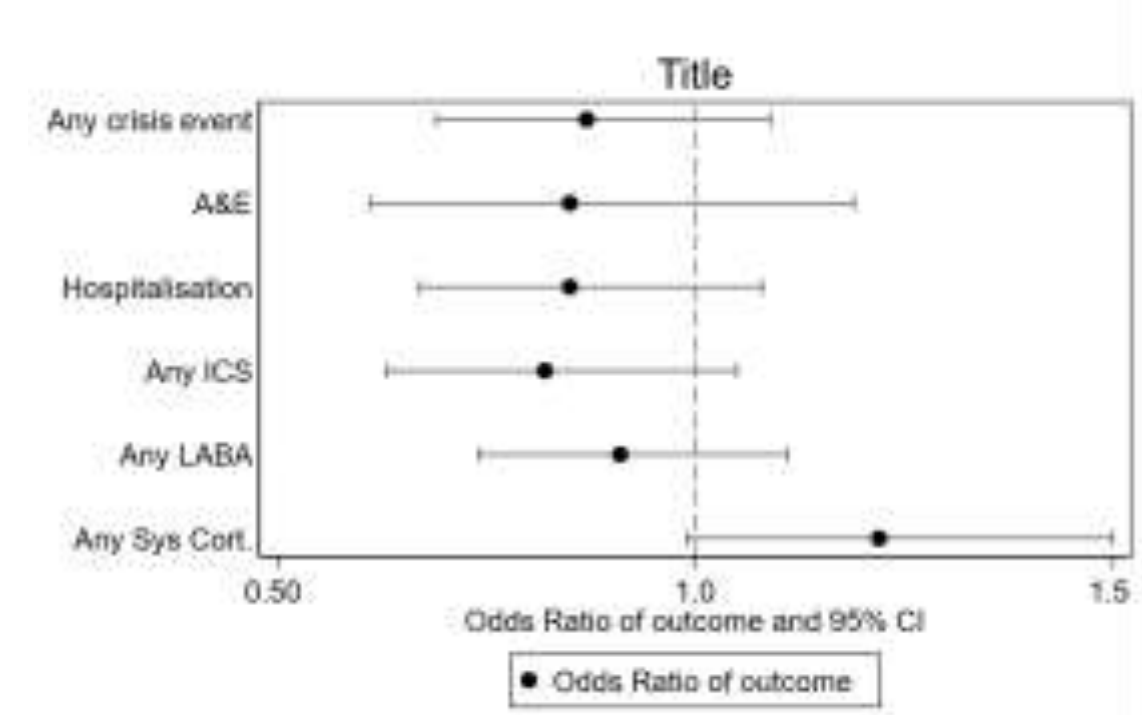

Thorax thorax.bmj.com @ThoraxBMJ British Thoracic Society BMJ Group
